# Correlation of SRSF1 and PRMT1 expression with clinical status of pediatric acute lymphoblastic leukemia

**DOI:** 10.1186/1756-8722-5-42

**Published:** 2012-07-27

**Authors:** Limin Zou, Han Zhang, Chaohao Du, Xiao Liu, Shanshan Zhu, Wei Zhang, Zhigang Li, Chao Gao, Xiaoxi Zhao, Mei Mei, Shilai Bao, Huyong Zheng

**Affiliations:** 1 Hematology Oncology Center, Beijing Key Laboratory of Pediatric Hematology Oncology, National Key Discipline of Pediatrics, Beijing Children’s Hospital, Capital Medical University, 56 Nanlishi Road, Beijing, 100045, China; 2 State Key Laboratory of Molecular Developmental Biology, Institute of Genetics and Developmental Biology, Chinese Academy of Sciences, West Beichen Road, Beijing, 100101, China

**Keywords:** Acute lymphoblastic leukemia, Splicing factor SRSF1, Protein arginine methyltransferase 1 (PRMT1), Alternative splicing, Arginine methylation

## Abstract

**Background:**

Acute lymphoblastic leukemia (ALL) is the most frequently-occurring malignant neoplasm in children, but the pathogenesis of the disease remains unclear. In a microarray assay using samples from 100 children with ALL, *SFRS1* was found to be up-regulated. Serine/arginine-rich splicing factor 1 (SRSF1, also termed SF2/ASF), encoded by the *SFRS1* gene, had been shown to be a pro-oncoprotein. Our previous study indicated that SRSF1 can be methylated by protein arginine methyltransferase 1 (PRMT1) in vitro; however, the biological function of SRSF1 and PRMT1 in pediatric ALL are presently unknown.

**Methods:**

Matched, newly diagnosed (ND), complete remission (CR) and relapse (RE) bone marrow samples from 57 patients were collected in order to evaluate the expression patterns of SRSF1 and PRMT1. The potential oncogenic mechanism of SRSF1 and PRMT1 in leukemogenesis was also investigated.

**Results:**

We identified significant up-regulation of SRSF1 and PRMT1 in the ND samples. Importantly, the expression of SRSF1 and PRMT1 returned to normal levels after CR, but rebounded in the RE samples. Our observation that SRSF1 could predict disease relapse was of particular interest, although the expression patterns of SRSF1 and PRMT1 were independent of the cytogenetic subtypes. In pre-B-cell lines, both SRSF1 and PRMT1 expression could be efficiently attenuated by the clinical chemotherapy agents arabinoside cytosine (Ara-c) or vincristine (VCR). Moreover, SRSF1 and PRMT1 were associated with each other in leukemia cells in vivo. Knock-down of SRSF1 resulted in an increase in early apoptosis, which could be further induced by chemotherapeutics.

**Conclusions:**

Our results indicate that SRSF1 serves as an anti-apoptotic factor and potentially contributes to leukemogenesis in pediatric ALL patients by cooperating with PRMT1.

## Background

Leukemia is the most frequent malignant neoplasm and one of the primary causes of death in children. The incidence rate of pediatric leukemia is 3-5/100,000 individuals, and nearly 15,000 children are newly diagnosed with leukemia in China each year. Acute lymphoblastic leukemia (ALL) accounts for 75 % of pediatric leukemia with peak levels of incidence from 2 to 5 years of age. In the last 30 years, optimal use of anti-leukemic agents in combination with chemotherapy regimens has improved the overall cure rate to 80 % in pediatric ALL [1]. However, up to 20 % of patients experience relapse [2], which ultimately results in treatment failure and death.

Serine-rich (SR) proteins are a family of RNA-binding proteins essential for diverse events during the life cycle of mRNAs, including transcription elongation, mRNA export, nonsense-mediated mRNA decay and translational regulation [3]. Alterations in mRNA expression frequently result in severe pathological consequences, and are often implicated in human diseases [4]. Serine/arginine-rich splicing factor 1 (SRSF1, also termed SF2/ASF), a prototypical SR protein encoded by the gene *SFRS1*, has been shown to be a potent oncoprotein and is up-regulated in many cancers [5]: it is an essential factor requisite in early constitutive splicing, acting as an alternative-splicing factor capable of influencing splice-site selection [6-8]. SRSF1 over-expression is sufficient to transform rodent fibroblasts and subsequently generate sarcomas in nude mice by controlling the alternative splicing of key tumor suppressors and oncogenes [5]. Although SRSF1 protein levels vary widely among cell types, tight control of SRSF1 abundance and activation appears significant for normal cellular and organismal physiology [9].

Post-transcriptional modification of RNA-binding proteins (RBPs) is primarily mediated by phosphorylation, acetylation, ubiquitination, SUMOylation and methylation, important mechanisms for fine-tuning the regulation of pre-mRNA splicing, export, stability, localization and translation [10,11]. Among these mechanisms, methylation processes are apparently deregulated in the emergence of several diseases [12,13]. It has become apparent in recent years that arginine residue methylation on proteins is involved in multiple cellular processes including regulation of transcription, RNA metabolism and DNA damage repair [14,15]. The most abundant methyltransferase in human cells is protein arginine methyltransferase 1 (PRMT1), which functions to monomethylate or asymmetrically dimethylate arginine residues. PRMT1 is well-established as an essential component of novel mixed lineage leukemia (MLL) oncogenic transcriptional complex [16]. In addition, RUNX1 (also termed AML1), one of the most important transcription factors in the regulation of mammalian hematopoiesis, is recognized to be arginine-methylated in vivo by PRMT1, indicating that PRMT1 serves as a transcriptional co-activator for RUNX1 function [17].

In our previous study, a total of 100 Chinese pediatric ALL bone marrow (BM) samples were studied utilizing the process of genome-wide microarray analysis [18,19]. Based on the dataset, we observed that the mRNA level of *SFRS1* (encoding SRSF1) was up-regulated in the leukemia cells. We recently reported that SRSF1 can be methylated by PRMT1 in vitro [20], which is consistent with findings that arginine methylation controls the subcellular localization and functions of SRSF1 [21]. To investigate the function of SRSF1 and PRMT1 in children with ALL, we detected the mRNA and protein expression levels of SRSF1 and PRMT1 at different stages of disease progression and demonstrated a similar pattern of SRSF1 and PRMT1 expression in ALL patient samples. The observation that SRSF1 can predict disease relapse in advance was significant. We also found that expression of SRSF1 and PRMT1 in the Nalm-6 (*TEL-AML1* positive) and Reh (*TEL-AML1* negative) cell lines could be attenuated with chemotherapy drugs; additionally, SRSF1 and PRMT1 were associated with each other in leukemia cells in vivo. Knock-down of SRSF1 resulted in early cell apoptosis. These data suggest that SRSF1 may contribute to the pathogenesis of ALL as an anti-apoptotic factor through an interaction with PRMT1, and SRSF1 may potentially represent a sensitive predictor of relapse.

## Methods

### Patient information

A total of 57 children (aged 7 months to 15 years, with a median age of 4 years) diagnosed with ALL between December 2002 and June 2011 were enrolled in this study, which took place in the Hematology Center of Beijing Children’s Hospital, Capital Medical University. Informed consent was obtained from all parents or legal guardians; a single sample was obtained from a child with idiopathic thrombocytopenic purpura (ITP) as a negative control. The study design followed Helsinki guidelines and was approved by the Beijing Children’s Hospital ethics committee of our hospital prior to initiating the study.

All patients were diagnosed with ALL using a combination of morphology, immunology, cytogenetics and molecular biology (MICM). The cytogenetic ALL subtypes were experimentally identified by G-banding karyotype and multiplex nested reverse-transcription-polymerase chain reaction (PCR). We tested for the presence of twenty-nine fusion genes, including *TEL-AML1*, *BCR-ABL*, *E2A-PBX1*, *MLL-AF4*, and *SIL-TAL1*.

Paired bone marrow (BM) samples from 45 pediatric patients (n = 90) were collected at the time they were characterized as newly-diagnosed (ND) and in complete remission (CR), from which 10 (n = 20) were selected for real-time PCR (RT-PCR) analysis, and another 35 (n = 70) were selected for western blot analysis. At the same time, unpaired BM samples from eight patients (n = 8) were collected, including 4 at ND and 4 in CR. In addition, the matched BM samples from an additional four relapsed patients were collected at the time of ND, CR and relapse (RE) (n = 12). The characteristics of these patients are described in detail in Additional files 1, 2 and 3.

### Cell samples, RNA isolation and quantitative Real-time PCR

The bone marrow samples were collected in ethylene-diaminetetraacetic acid (EDTA) tubes. Mononuclear cells were isolated from diagnostic BM samples by Ficoll gradient centrifugation (MD Pacific, Tianjin, China, density: 1.077g/ml) after which they were cryo-preserved in a −80°C freezer. Total RNA was extracted using Trizol reagent according to the manufacturer’s instructions (Invitrogen, Paisley, UK). cDNA was synthesized using random hexamers and Moloney murine leukemia virus reverse transcriptase (Promega, Madison, USA). Real-time PCR was performed on a Mastercycler ep Realplex2 (Eppendorf, Germany) using SYBR Green fluorescence in accordance with manufacturer’s instructions (RealMasterMix Kit, TIANGEN, Beijing, China), using *GAPDH* gene as an internal control. The primer sequences were as follows: *SFRS1*, 5′-GATTACGATGGGTACCGTCTGC-3′ and 5′-GCAGTCCAGAGACAACCACTC-3′; *PRMT1*, 5′-GATGCTGAAGGACGAGGTGC-3′ and 5′-ACTCGATCCCGATGACCTTGCG-3′; *GAPDH*, 5′-GGTCGGAGTCAACGGATTTGG-3′ and 5′-CATGGAATTTGCCATGGGTGGAATC-3′. The initial denaturation was performed at 95°C for 1 minute, followed by 45 cycles of 10s at 95°C, 10s at 55°C and 15s at 68°C. The PCR products were analyzed using the Realplex software. In order to monitor reproducibility and reliability, each assay was repeated three times. Stringent measures to prevent sample contamination included three non-template negative controls (NTC- reaction mix without DNA, and distilled water alone).

### Plasmid construction and preparation

The U6 promoter-driven shRNA expression vector pNeoU6+1 and the shRNA plasmid specific for firefly luciferase (sh-luc) had been prepared in advance in our lab facility. Both plasmids contained a GFP tag. The two target sites in the SRSF1 mRNA coding regions were sh-SRSF1-1 (62–81, GTAACTTACCTCCAGACATC) and sh-SRSF1-2 (270–289, AAGCGGCCGTGGAACAGGCC). The single shRNA targeting the PRMT1 mRNA coding region was sh-PRMT1 (379–399, GTGAAGATCGTCAAAGCCAAC). These targeted sequences were verified in the human genomic and transcriptional sequence database (NCBI) as unique sequences. The plasmids were purified using a Plasmid Mini Kit (Omega, Bio-tek) in accordance with manufacturer’s instructions.

### Cell culture and drug treatment

Nalm-6 is a pre-B ALL cell line with no fusion gene, while Reh is a pre-B cell line with the *TEL-AML1* fusion gene. The Normal B (NB) cell line is derived from Epstein-Barr-virus (EBV)-transformed human B cells. Nalm-6, Reh and NB cells were cultured in a modified HyQ RPMI-1640 medium (Hyclone) which was supplemented with 10% fetal bovine serum (FBS) (PAA) in a 5% CO_2_ humidified atmosphere at 37°C. For the clinical chemotherapeutic induction experiments in the leukemia cell lines, 1×10^7^ cells were treated with 10μg vincristine (VCR, Shenzhen Main Luck Pharmaceuticals), 500 μg cytarabine (Ara-c, Pharmacia & Upjohn) or 50 μl normal saline (NS) in 10ml of fresh media containing 10%FBS for 24 hours or 48 hours. Cell lines were harvested at 24 and 48 hours after drug treatment, respectively, and siRNA-treated cells were harvested at 72 hours after transfection for western blot analysis.

The cells were washed three times with PBS, then incubated on ice for 30 min in a 1× cell lysis buffer [20 mM Tris, 50 mM NaCl, 2 mM Na_3_VO_4_, 10mM NaF, 1mM EDTA, 0.1% Triton X-100, and Proteinase Inhibitor Cocktail (Roche)], then sonicated. Following centrifugation at 4°C for 30 minutes, the supernatants were frozen at −80°C or used immediately.

### Western blot and antibodies

Samples containing 20 μg of total protein were separated on 12% SDS-PAGE gels and then transferred onto nitrocellulose membranes (Whatman) in transfer buffer (25 mM Tris-base, 40 mM glycine, and 20% methanol) using the Mini Trans-Blot Cell (BIO-RAD) at 400 mA for 3 hours. The membranes were blocked by incubation with 5% nonfat milk in TBS-T (20 mM Tris [pH 7.6], 137mM NaCl, and 0.1% Tween 20) for 1 hour at room temperature. Proteins were detected using specific mouse monoclonal anti-SRSF1 (1:2,000, Santa Cruz, CA, USA), anti-PRMT1 (1:2,000, Sigma) or rabbit monoclonal anti-GAPDH (1:5,000, prepared in our lab) antibodies. After washing with TBS-T, the membranes were incubated with goat anti-mouse or goat anti-rabbit immunoglobulin G secondary antibodies (1:5,000, Pierce) in TBS-T containing 5% nonfat milk for 45 min at room temperature. The proteins were visualized using an enhanced chemi-luminescence kit (Amersham). The membranes were stripped by incubation in stripping buffer (62.5 mM Tris-base, 2% SDS, and 100 mM 2-mercaptoethanol), then blocked and probed as described above.

### Semi-quantitative analysis

Semi-quantitative analysis based on the western blot was performed using Gel-pro analyzer 4.0 software [22]. The relative expression level of SRSF1 or PRMT1 was normalized by integrated optical density (IOD) of SRSF1 or PRMT1, against that of GAPDH (loading control).

### Cell apoptosis assay

The shRNA plasmids were transfected into Nalm-6 cells using the Amaxa Cell Line Nucleofector Kit T and Nucleofector Device (Lonza) according to manufacturer instructions, after which the cells were incubated in for 72 hours, in 2 millileters of antibiotic-free media containing 10 % FBS. GFP-positive cells were sorted by flow cytometry (BD, FACSAria II) and collected in order to measure silencing efficiency. For the apoptosis assay, cells were treated with VCR, Ara-c or NS at 48 hours after transfection, after which they were harvested at 72 hours for apoptosis analysis. The apoptotic cell death was evaluated using annexin V-APC/PI staining (BD) and flow cytometry in accordance with manufacturer instructions.

### Immune-precipitation and co-immune-precipitation assays

Leukemia cell extracts were prepared using RIPA buffer [100 mM NaCl, 20 mM NaH_2_PO_4_ (pH = 7.4), 10 mM NaF, 2 mM Na_3_VO_4_, 1.0 % NP40, Proteinase Inhibitor Cocktail (Roche) and 10 mM PMSF], and 1.0 μg of anti-SRSF1 or anti-PRMT1 antibodies were used for binding overnight at 4 °C, to perform immune-precipitation from 1.0 milligrams of cell lysates. The Protein G-Plus Beads (Calbiochem) were then added to the reaction system for binding, in a cold room, for a period of 2 hours. The immune-precipitates were washed three times with washing buffer [150 mM NaCl, 20 mM NaH_2_PO_4_ (pH = 7.4), 10 mM NaF, 2 mM Na_3_VO_4_, 1.0 % NP40 and 10 mM PMSF] for a duration of 10 minutes per wash. The immune-precipitates were then detected by standard western blot analysis as described above.

## Results

### Expression of both the splicing factor SRSF1 and PRMT1 in clinical samples

**Figure 1:**
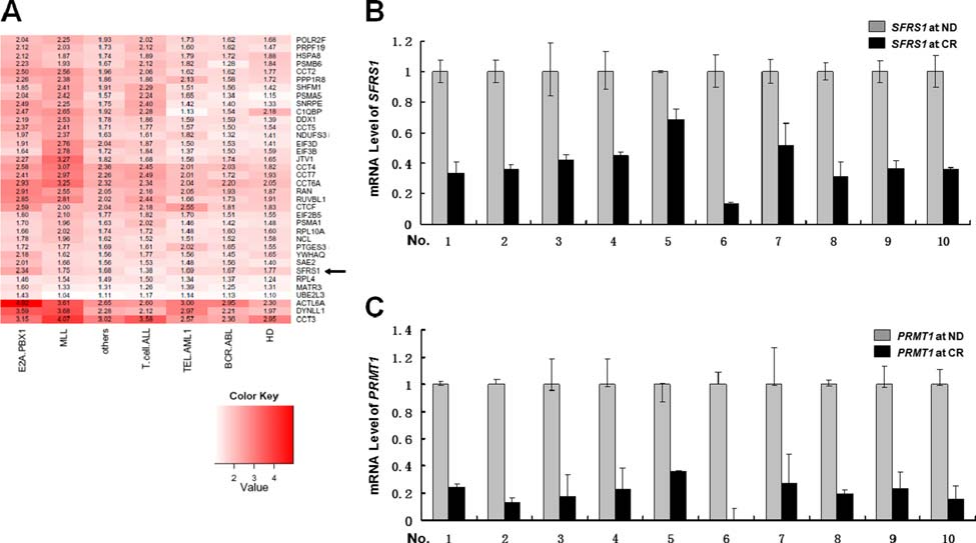
**The mRNA levels of SRSF1 and PRMT1 are dramatically up-regulated in pediatric ALL cases.** (**A**) Heat map of the mRNA level of *SFRS1* (black arrow). The fold changes of the differential expressions compared with the control were indicated by the color intensity with red representing up-regulation. HD, hyperdiploid > 50 chromosomes. (**B**) The mRNA level of *SFRS1* was measured by RT-PCR in paired cDNA samples from 10 ALL patients (n = 20). Each paired sample refers to two samples from one patient at the time of ND and CR, respectively. The *SFRS1* mRNA level was higher in the ND samples compared to the CR samples (fold change 2.53, *p* = 0.000, Paired Samples *T* test). (**C**) The mRNA level of *PRMT1* was measured by RT-PCR in paired cDNA samples from 10 ALL patients. The *PRMT1* mRNA level was dramatically higher in the ND samples compared to CR samples (fold change 4.95, *p* = 0.000, Paired Samples *T* test). ND, newly diagnosed. CR, complete remission.

In addition to the results of our previous study using genome-wide microarray analysis from 100 pediatric ALL cases [18,19], we further found that *SFRS1* (encoding SRSF1) is up-regulated in leukemia cells (Figure 1A and Additional file 4). To validate this finding, RT-PCR analysis was performed to verify the transcriptional level of *SFRS1* in 10 paired cDNA samples (n = 20). Each paired sample refers to two samples from one patient at the time of newly diagnosed (ND) and complete remission (CR), respectively. The mRNA level of *SFRS1* was elevated in ND samples compared with CR samples (Figure 1B, fold change 2.53, *p* = 0.000, Paired Samples *T* test), which was consistent with the bio-informatics analysis (Figure 1A).

We recently reported that PRMT1 can specifically methylate SRSF1 in vitro [20]. We therefore examined the expression pattern of PRMT1 in leukemic cells. Similar to the RT-PCR results obtained for SRSF1, the mRNA level of *PRMT1* was significantly elevated in ND samples compared with CR samples (Figure 1C, fold change 4.95, *p* = 0.000, Paired Samples *T* test).

**Figure 2:**
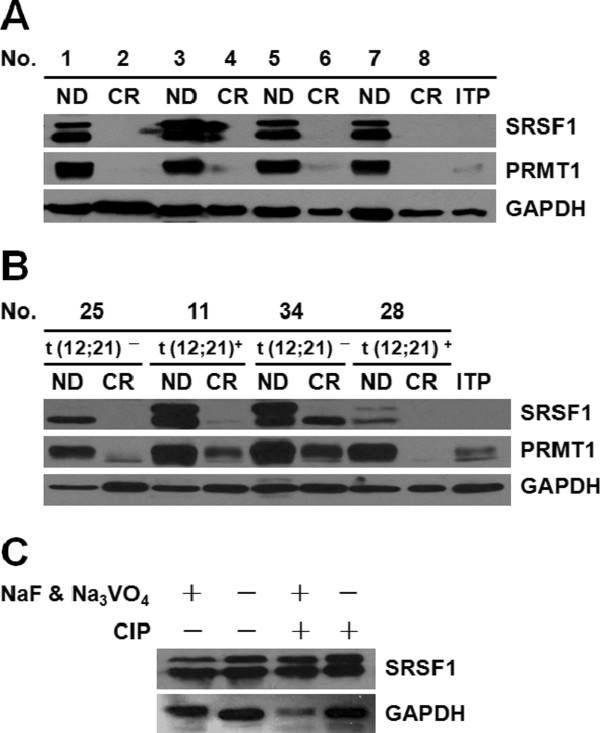
**The expression levels of SRSF1 and PRMT1 are dramatically up-regulated in pediatric ALL.** (**A**) Western blot analysis of unpaired samples from 8 patients (n = 8, 4 ND and 4 CR). Both SRSF1 and PRMT1 were over-expressed in ND samples, and displayed reduced levels of expression after CR. A sample from one pediatric ALL patient with idiopathic thrombocytopenic purpura (ITP) was used as a negative control, and GAPDH was used as a loading control. (**B**) Western blot analysis was of paired samples taken from 35 pediatric ALL patients (n = 70). The results from 4 such patients are shown here, including 2 with t(12;21) (*TEL-AML1*) translocation and 2 with no translocation. Both SRSF1 and PRMT1 were over-expressed in ND samples and displayed reduced levels of expression after CR. (**C**) Eliminating NaF and Na_3_VO_4_ from the lysis buffer and adding calf intestinal alkaline phosphatase (CIP) resulted in no change in the upper bands of SRSF1.

To investigate the translational levels of SRSF1 and PRMT1, western blot analysis was performed to measure the protein levels of SRSF1 and PRMT1 in samples from 43 patients, including 8 unpaired samples (n = 8, 4 ND and 4 CR) and 35 paired samples (n = 70). One BM sample from a patient with idiopathic thrombocytopenic purpura (ITP) was selected as a negative control. Both SRSF1 and PRMT1 were over-expressed in ND samples, and were reduced to normal levels upon CR (Figure 2A and 2B), which is supported by reports that SRSF1 and PRMT1 are highly expressed in many cancers.

Interestingly, the western blot analysis in clinical samples indicated that the SRSF1 protein could appear as upper and/or lower bands. Since the phosphorylation status of SRSF1 is critical for its normal function [23,24], we eliminated Na_3_VO_4_ and NaF from the lysis buffer and treated the samples with calf intestinal alkaline phosphatase (CIP). Results revealed no changes in the upper bands, suggesting that these bands are not phosphorylation forms of SRSF1 (Figure 2C); of interest because SRSF1 has been reported to be auto-regulated into multiple isoforms that differ in function in various physiological or pathological conditions [25]. Therefore, although the upper band might be an isoform of SRSF1 with distinct functions, this hypothesis requires further investigation.

### Expression patterns of SRSF1 and PRMT1 are not associated with different cytogenetic abnormalities in clinical samples

Final clinical results are related to the hematological, immunological and molecular genetic features identified at the time of patient diagnosis [26]. Patients with hyperdiploidy or a TEL-AML1 translocation usually present with low-risk clinical features and thus have favorable long-term remission and survival rates; patients with a BCR-ABL or MLL translocation tend to have high leukemic burdens and poorer prognosis [27-29]. Given these differences in outcome among patients with varying cytogenetic abnormalities, we aimed at determining whether SRSF1 and PRMT1 expression signatures characterize these subgroups.

**Figure 3:**
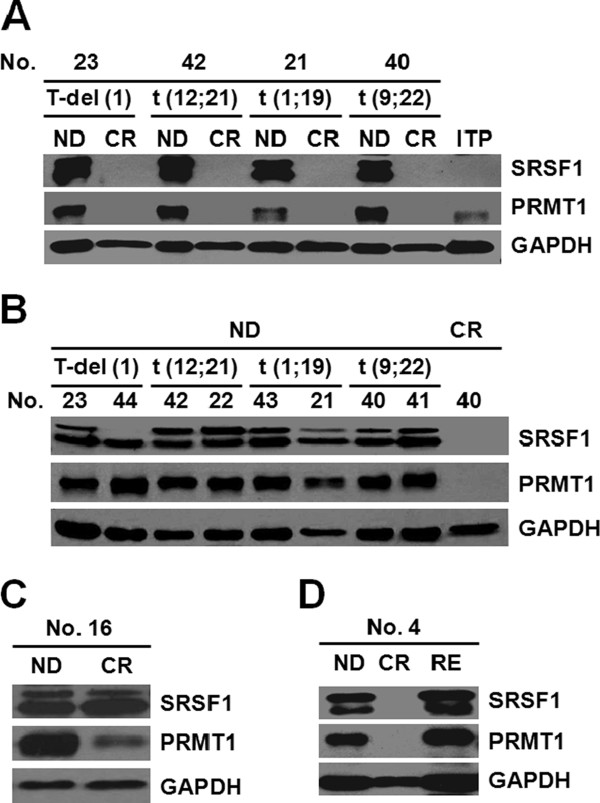
**Expression of SRSF1 and PRMT1 among different cytogenetic subtypes and relapsed ALL patients.** (**A**) The expression of SRSF1 and PRMT1 in paired samples from different cytogenetic subtypes was detected, including T-cell ALL with deletion of chromosome 1 [T-del(1)], t(12;21) (*TEL-AML1*), t(1;19) (*E2A-PBX1*) and t(9;22) (*BCR-ABL*), and no obvious difference was found among these subtypes. (**B**) No obvious change was found in ND samples among these subtypes. (**C**) The over-expression of SRSF1 in the CR phase in a rare case (No. 16), who experienced an isolated CNS relapse 8 days after the collection of CR sample. (**D**) Both SRSF1 and PRMT1 expression rebounded after relapse, but remained at a normal level in the CR phase. Four relapsed cases were detected, with the result from case No. 4 being shown.

We first detected the effect of the t(12;21) (*TEL-AML1*) translocation, the most common chromosomal translocation in pediatric B-ALL, on the expression of both proteins in paired clinical samples. Over-expression of both proteins was observed in the ND phase, but the expression levels declined to normal levels upon CR. No differences in the specific features were observed between the two groups (Figure 2B). We subsequently assessed paired samples from different subtypes of ALL, including T-cell ALL with deletion of chromosome 1 [T-del(1)], t(12;21) (*TEL-AML1*), t(1;19) (*E2A-PBX1*) and t(9;22) (*BCR-ABL*), and we found similar results among the different subtypes (Figure 3A). Moreover, no differences were observed in the expression patterns of both proteins in ND samples from different subtypes of ALL (Figure 3B). Abundant data indicate that both SRSF1 and PRMT1 display similar expression patterns with no specific features in both paired samples and ND samples that are independent of the cytogenetic subtypes.

### SRSF1 might be a predictor of relapse and remission

Notably, one specific case among the 35 paired ALL cases displayed a SRSF1 level much higher in the CR phase compared with other CR samples, while the PRMT1 expression level remained low (Figure 3C). In reviewing the clinical information for this patient we discovered that he experienced an isolated CNS relapse 8 days after collection of the CR sample, although no other clinical findings had predicted a relapse. This observation suggested that SRSF1 may be a sensitive predictor of relapse. SRSF1 has been shown to enhance transformation in some cancers [5]; therefore, the increase in the level of SRSF1 should occur earlier than the morphological or immunological change.

To investigate whether samples from relapsed ALL patients share the same feature with this patient, additional samples from four relapsed ALL patients (who relapsed at least 30 months after CR) were collected. In all four cases, both SRSF1 and PRMT1 expression increased after relapse, but none displayed an abnormally high level at CR (Figure 3D). These results demonstrated that although SRSF1 expression most likely reflects a short-term state prior to relapse, it does not promote the ALL relapse.

### SRSF1 and PRMT1 can be induced by chemotherapeutic drugs in ALL cell lines

**Figure 4:**
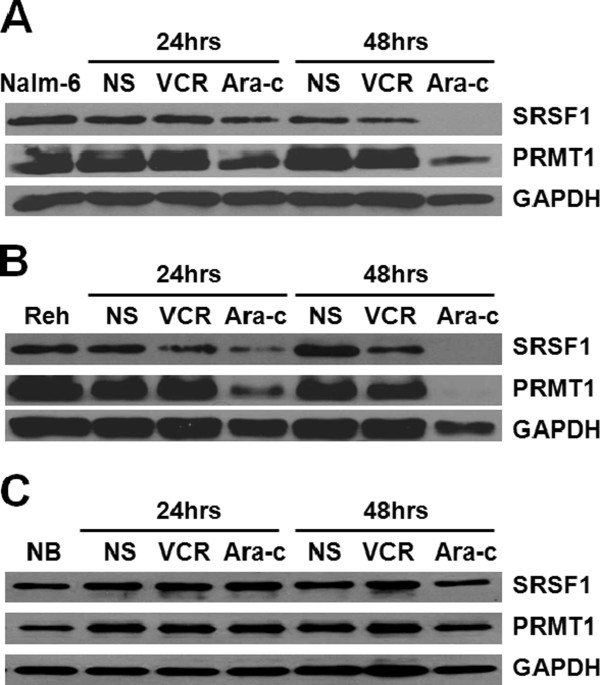
**Expression of SRSF1 and PRMT1 in cell lines before and after treatment with chemotherapeutics.** Nalm-6 cells (**A**), Reh cells (**B**) and Normal B (**C**) cells were treated with VCR, Ara-c and NS (negative control) for 24 and 48 hours, respectively. Cell lysates were probed with anti-SRSF1 and anti-PRMT1 antibodies, and GAPDH was used as a loading control. Nalm-6 is a pre-B ALL cell line with no fusion gene, while Reh is a pre-B cell line with the *TEL-AML1* fusion gene. The normal B (NB) cell line is derived from EBV-transformed human B cells.

The *TEL-AML1* fusion gene is generated by the t(12;21) (p13;q22) translocation, which accounts for 25 % of pediatric B-ALL with favorable prognosis. Given the complicated influence of other genetic abnormalities in BM samples, we selected ALL cell lines with which to investigate the effects of chemotherapeutics drugs on the expression of SRSF1 and PRMT1. The Reh cell line carries the *TEL-AML1* fusion gene, whereas the Nalm-6 cell line contains no fusion gene; the Normal B (NB) cell line was used as a control. To compare expression changes in SRSF1 and PRMT1 before and after treatment with chemotherapeutics, all cell lines were treated with either vincristine (VCR), cytarabine (Ara-c) or normal saline (NS) for both 24 and 48 hours. The expression levels of both proteins decreased 48 hours after treatment with VCR and Ara-c in Nalm-6 and Reh cells; SRSF1 and PRMT1 were more sensitive to Ara-c than VCR (Figure 4A and 4B). In contrast, nearly no expression changes were observed before and after treatment in the NB cell line (Figure 4C). The effect of the chemotherapeutics appeared to be more prominent in Reh cells than in Nalm-6 cells, indicating that the *TEL-AML1* fusion gene contributed to a positive response, which was in agreement with clinical responses to chemotherapy.

### SRSF1 and PRMT1 are associated with each other in vivo

**Figure 5:**
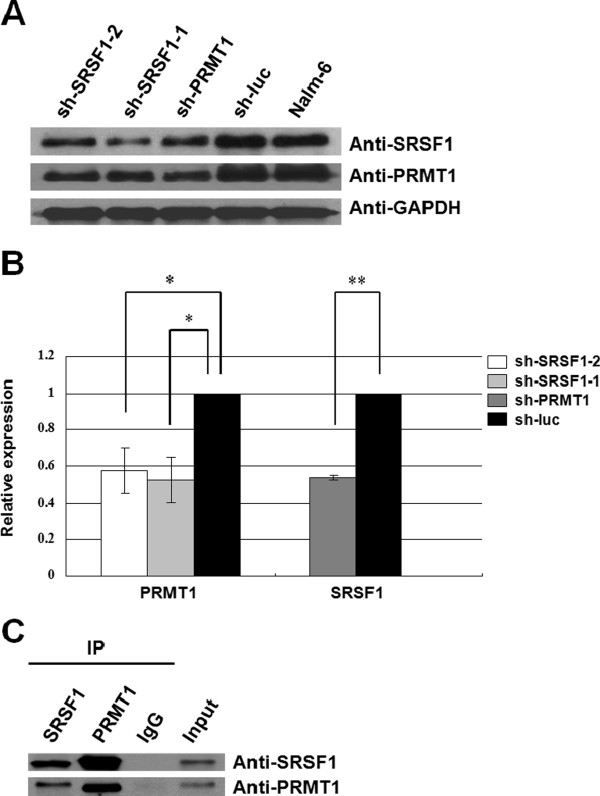
**Interaction between SRSF1 and PRMT1.** (**A**) Knock-down of SRSF1 or PRMT1 could reciprocally down-regulate each other. Both sh-SRSF1-1 and sh-SRSF1-2 are shRNA plasmids specific for SRSF1. sh-PRMT1 is the shRNA plasmid specific for PRMT1. sh-luc is the shRNA plasmid specific for firefly luciferase. sh-luc and untreated Nalm-6 cells were used as negative controls. (**B**) The expression of SRSF1 and PRMT1 were semi-quantified by Gel-pro analyzer software based on the western blot. The alterations of their expression levels were statistically analyzed. The knock-down of SRSF1 by sh-SRSF1-1 and sh-SRSF1-2 resulted in 1.90-fold (*p* = 0.022) and 1.73-fold (*p* = 0.028) decreases in the expression of PRMT1, respectively. The knock-down of PRMT1 by sh-PRMT1 resulted in a 1.86-fold decrease in the expression of SRSF1 (*p* = 0.000). (**C**) A co-immuno-precipitation assay was performed in Nalm-6 cells, and the results indicated that endogenous SRSF1 and PRMT1 can associate with each other in vivo. An IgG antibody was used as a negative control.

Based on the western blot analysis of clinical samples, SRSF1 and PRMT1 shared a similar pattern of expression during the disease process. We have previously shown that SRSF1 can be methylated by PRMT1 in vitro [20]. In order to determine whether these proteins interact with each other, we first knocked-down either SRSF1 or PRMT1 and detected their expression levels in Nalm-6 cells. After successful interference, the second protein was also down-regulated (Figure 5A). The parallel semi-quantitative analysis revealed that the knock-down of SRSF1 by sh-SRSF1-1 and sh-SRSF1-2 resulted in 1.90-fold and 1.73-fold decreases in the expression of PRMT1, respectively (Figure 5B*p* = 0.022 and *p* = 0.028, respectively); similarly, the knock-down of PRMT1 by sh-PRMT1 resulted in a 1.86-fold decrease in the expression of SRSF1 (Figure 5B*p* = 0.000), indicating that SRSF1 and PRMT1 exhibit mutual dependency on their respective stabilities.

To further investigate the relationship between these two proteins in vivo, immune-precipitation (IP) was performed using anti-SRSF1 or anti-PRMT1 antibodies in whole cell lysates of the Nalm-6 cell line. The precipitates were analyzed using both PRMT1 and SRSF1 antibodies. Reciprocal co-IP was performed, with results indicating that endogenous SRSF1 and PRMT1 exist in one complex, and that they physiologically associate with each other in leukemic cells in vivo (Figure 5C).

### SRSF1 plays an anti-apoptotic role in chemotherapy process

**Figure 6:**
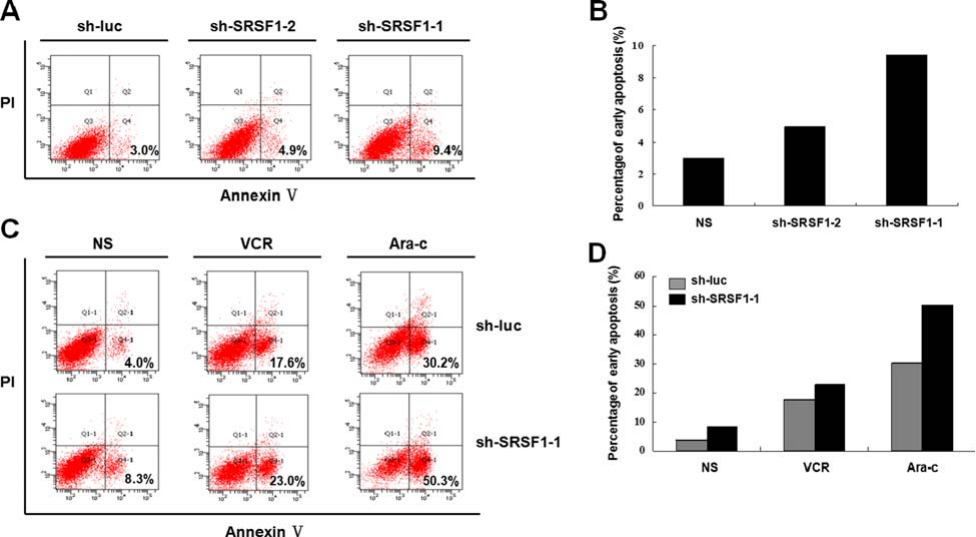
**SRSF1 plays an anti-apoptotic role in the response to chemotherapy.** (**A**) The knock-down of SRSF1 resulted in an increase in early cell apoptosis. The percentages of annexin V(+)/PI (−) cells are indicated in the figure. sh-luc (targeting firefly luciferase) was used as a control. (**B**) The percentage of early apoptosis in each group is shown in the diagram. The knock-down of SRSF1 by sh-SRSF1-1 resulted in a higher percentage of early cell apoptosis. (**C**) Nalm-6 cells were treated with VCR, Ara-c or NS (negative control) at 48 hours after transfection with the shRNA plasmids. The cellular apoptosis was assessed at 72 hours after transfection by FACS analysis of annexin V-APC/PI staining. The percentages of annexin V(+)/PI (−) cells are indicated in the figure. (**D**) The diagram indicates the percentage of early apoptosis in each group. The knock-down of SRSF1 by sh-SRSF1-1 resulted in a further increase in early cell apoptosis induced by VCR or Ara-c.

The high expression of the splicing factor SRSF1 in leukemic cells prompted us to investigate whether SRSF1 might affect apoptosis in lymphoblastic cells. To assess the effect of SRSF1 on cell apoptosis, two SRSF1 shRNA-expressing plasmids (sh-SRSF1-1 and sh-SRSF1-2) were constructed. The shRNA plasmid specific for firefly luciferase (sh-luc) was used as a control. The RNA interference efficiency of both shRNAs was evaluated by western blot in Nalm-6 cells (Figure 5A), which showed a better knock-down efficiency of sh-SRSF1-1 than sh-SRSF1-2. Cellular apoptosis was detected at 72 hours after transfection of the shRNA plasmids. Because all of the plasmids carried a GFP tag, we detected cell apoptosis in GFP-positive cells sorted by flow cytometry. Knock-down of SRSF1 by sh-SRSF1-1 and sh-SRSF1-2 resulted in 3-fold and 1–2-fold increases in early apoptosis, indicating that SRSF1 is an anti-apoptotic factor which further validated the superior knock-down efficiency of sh-SRSF1-1 compared to sh-SRSF1-2 (Figure 6A and 6B); sh-SRSF1-1 was selected to perform the subsequent experiments.

To investigate the effects of chemotherapeutic drugs on cell apoptosis, we further introduced VCR, Ara-c or NS into Nalm-6 cells at 48 hours after transfection of the shRNA plasmids. Cellular apoptosis was examined at 72 hours after transfection. Both VCR and Ara-c induced apoptosis in Nalm-6 cells, while knock-down of SRSF1 by sh-SRSF1-1 further increased the proportion of cells undergoing early apoptosis induced by VCR or Ara-c (Figure 6C and 6D). These results demonstrated that SRSF1 plays an anti-apoptotic role in the chemotherapy process.

## Discussion

Splicing factor SRSF1 is a key member of the SR protein family. SRSF1 has been identified as an oncoprotein involved in many cancers, including those of the lung, colon, breast, as well as in hepatocellular carcinoma [30]. Over-expression of SRSF1 is sufficient to cause transformation of fibroblasts by controlling alternative splicing of tumor suppressors and oncogenes [5]. Until now, there has been no relevant report of SRSF1 in leukemia cells. Based on our previous genome-wide microarray analysis of samples from 100 children with ALL, we further found over-expression of *SFRS1* at the mRNA level, which prompted us to explore the biological function of SRSF1 in pediatric ALL.

In this study, we collected samples from 43 pediatric ALL patients (35 paired and 8 unpaired BM samples), and found that both the mRNA and protein levels of SRSF1 were up-regulated in ND samples and returned to normal levels in CR samples after chemotherapy. In ALL cell lines, SRSF1 could be down-regulated by VCR and Ara-c treatment, which are commonly used in the clinical chemotherapy of ALL. These findings suggest that SRSF1 may represent a promising indicator of disease progression as well as reflecting the ongoing effects of treatment.

Over the past 50 years, pediatric ALL treatment has evidenced some of the most dramatic cancer success stories. ALL has transitioned from the status of an untreatable terminal diagnosis to becoming a treatable disease [31]. Unfortunately, the overall cure rate of pediatric ALL has not achieved a significant increase in recent years: approximately 20 % of patients relapse, which is a leading factor in treatment failure which dramatically reduces a long-term, disease-free survival rate. Currently, morphology, immunology, cytogenetics and molecular biology (MICM) remain the key methods for the evaluation of pediatric ALL relapse and risk-stratification for treatment. Patients with a t(9;22) translocation have a high risk of relapse, but this translocation accounts for only 3 % of pediatric ALL cases. However, no specific relapse markers have been found in other subgroups of pediatric ALL. Notably, this study recently revealed the identification of SRSF1 expression signatures associated with the timing of relapse. One specific case among the 35 ALL paired samples displayed an SRSF1 expression level was substantially elevated in the CR phase; clinical data revealed that this patient suffered an isolated CNS relapse 8 days after collection of the CR sample, yet no indications of the approaching relapse had been observed. Such a rare case indicated that the level of SRSF1 had been altered in advance, and was a more sensitive and earlier predictor of relapse than other morphological and immunological criteria. Conversely, this patient achieved a complete hematologic remission (a state of basically normal complete blood count, with no blasts in the peripheral blood and less than 5 % blasts in the BM) under chemotherapy. However, the treatment did not effectively reduce the SRSF1 level, which could point to a relapse-driving event. To further explore this issue, four relapsed ALL patients were enrolled to observe the changes in expression of SRSF1 during different phases. We found that SRSF1 increased again upon disease relapse, but it remained at a normal level in CR samples (which were extracted more than one year before relapse). This finding indicates that the level of SRSF1 increased as the malignant clones expanded, being detectable only a short time in advance of disease recurrence. We were fortunate to observe such an unusual event, and to obtain this rare CR sample. Additional clinical samples must be further studied and results verified if we are to determine the exact time of SRSF1 up-regulation in advance of disease recurrence.

*TEL-AML1* is the most common chimeric fusion gene in pediatric B-ALL. We first compared the expression changes in SRSF1 between *TEL-AML1*-positive and -negative groups in clinical samples, but no differences were found. Further experiments showed a similar expression pattern of SRSF1 among the different subgroups. These results indicate that the expression pattern of SRSF1 is independent of molecular biological differences. However, the drug-induced experiment showed a more dramatic down-regulation of SRSF1 by VCR and Ara-c in Reh cells than in Nalm-6 cells, consistent with the clinical response, suggesting that *TEL-AML1* is a marker of a favorable prognosis. This contrast suggested that additional genetic abnormalities in clinical samples and multiple chemotherapy agents were involved in treatment. Therefore, influencing factors in clinical samples are more complex, while the simplified conditions in cell lines and single drug treatments could allow the results to be more persuasive. Further clinical observations are clearly required for understanding the results of these studies.

In recent years, protein arginine methylation has been detected on abundant functional proteins, such as histones, RNA processing proteins, DNA repair proteins and signal transduction proteins [14]. Arginine methyltransferases are a group of enzymes that transfer methyl groups from S-Adenosylmethionine (SAM) to the guanidinoside chain of arginine residues. PRMT1 is the most abundant arginine methyltransferase in human cells and has been linked to some cancers, including MLL, with varying expressions of every isoform [16,32-35]. PRMT1 expression is up-regulated when CD34^+^ cells are stimulated to differentiate into myeloid cells in in vitro cultures [11]. In our previous study, we reported that PRMT1 can methylate SRSF1 at R93, R97 and R109 residues in the G-Hinge region in vitro. We therefore examined the expression signature of PRMT1 in clinical BM samples and cell lines. Here, we observed an expression pattern of PRMT1 that was similar to SRSF1, except for a specific case in which the PRMT1 level remained lower in the CR phase. This difference indicated that alterations in PRMT1 lag behind SRSF1 in relapsed ALL cases.

PRMT1 has been reported to directly methylate RUNX1, a critical transcription factor involved in approximately 30 % of pediatric leukemia cases. Together with PRMT1 up-regulation, RUNX1 and methylated RUNX1 levels are also up-regulated. The interaction between PRMT1 and RUNX1 facilitates differentiation by remodeling the chromatin structure for lineage-specific genes [17]. In addition, SR proteins undergo extensive post-translational modifications, which have been shown to play a key role in modulating protein-protein and protein-RNA interactions within the spliceosome. In this study, we found that the SRSF1 or PRMT1 expression levels could be influenced by each other in a leukemia cell line. Further data showed that SRSF1 could physically associate with PRMT1 in vivo. Therefore, the interaction between these proteins may have oncogenic functions in leukemogenesis.

Leukemia is recognized as a progressive, malignant disease caused by distorted differentiation, apoptosis and proliferation of hematopoietic cells at different stages. Here, we found that the knock-down of SRSF1 increased the early apoptosis of leukemia cells. Further treatment with the anti-leukemic drugs VCR and Ara-c in leukemia cells resulted in an increase in early apoptosis, which indicated that SRSF1 plays an anti-apoptotic role in chemotherapy. Moreover, the knock-down of SRSF1 increased the sensitivity of leukemia cells to the chemotherapy agents, suggesting that SRSF1 may be a potential target for anti-leukemic therapy.

## Conclusions

We first linked SRSF1 with pediatric ALL. We observed that the expression level of SRSF1 could serve as a sensitive indicator of CR and RE in ALL. As an anti-apoptotic factor, over-expression of SRSF1 causes the evasion of apoptosis in leukemic cells, while the knock-down of SRSF1 increases the sensitivity of leukemia cells to the chemotherapy agents, indicating that SRSF1 could potentially become a target for anti-leukemic therapy. Furthermore, the interaction between oncoprotein SRSF1 and PRMT1 may potentially contribute to leukemogenesis. In future studies, we will investigate additional samples to elucidate the role of SRSF1 in pediatric ALL.

## Competing interests

The authors declare that they have no competing interests.

## Authors’ contributions

LZ carried out the detection of all clinical samples by western blot, and participated in cell culture and drug treatment experiments; H Zhang performed quantitative RT-PCR, cell apoptosis assays, semi-quantitative analysis, co-immunoprecipitation assays and participated in cell culture and drug treatment experiments. Both LZ and H Zhang were involved in data analysis, drafted the manuscript and contributed equally in this study; CD performed shRNA plasmids construction, and participated in drafting the manuscript; XL participated in cell apoptosis assays; SZ carried out the bio-informatics analysis and participated in drafting the related method; WZ produced the heat map; ZL and CG collected the clinical ALL samples; XZ performed RNA isolation and cDNA synthesis; MM participated in the bio-informatics analysis; SB conceived the idea of the study and participated in its design; H Zheng guided the research, participated in the study design, and revised the manuscript. All authors read and approved the final manuscript.

## Supplementary Material

Additional file 1 **Table S1.** Clinical features of the pediatric acute leukemia cases for the paired bone marrow samples. Detailed characteristics of 45 pediatric ALL patients for the paired samples are indicated here. Patient No. 16 experienced an isolated CNS relapse 8 days after the collection of the CR sample; sadly, he died 1 month later following CNS relapse. Following admission to a different hospital, patient No. 38 received treatment with a full dose of dexamethasone for a period of 4 days, resulting in a low level of blasts in bone marrow, which blocked the immune-phenotype analysis. Click here for file

Additional file 2 **Table S2.** Clinical features of the pediatric acute leukemia cases for the unpaired bone marrow samples. Detailed characteristics of eight pediatric patients for the unpaired samples are shown here. Click here for file

Additional file 3 **Table S3.** Clinical features of pediatric acute leukemia cases for the relapsed bone marrow samples. Detailed characteristics of four relapsed patients are shown here. Click here for file

Additional file 4 **Doc1.** Bio-informatics methods for the heat map of mRNA level of*SFRS1*. Detailed methods of bio-informatics analysis of mRNA level of *SFRS1* are shown here [18,19][36]. Click here for file
