# Supplementary material for: Correlation of SRSF1 and PRMT1 expression with clinical status of pediatric acute lymphoblastic leukemia
Source: J Hematol Oncol. 2012 Jul 27;5:42. doi: 10.1186/1756-8722-5-42 (PMC3459738; doi:10.1186/1756-8722-5-42)
Supplement: Additional file 1 — Table S1. Clinical features of the pediatric acute leukemia cases for the paired bone marrow samples. Detailed characteristics of 45 pediatric ALL patients for the paired samples are indicated here. Patient No. 16 experienced an isolated CNS relapse 8 days after the collection of the CR sample; sadly, he died 1 month later following CNS relapse. Following admission to a different hospital, patient No. 38 received treatment with a full dose of dexamethasone for a period of 4 days, resulting in a low level of blasts in bone marrow, which blocked the immune-phenotype analysis. [file 1756-8722-5-42-S1.docx]

**Additional file 1.** **Clinical features of the pediatric acute leukemia cases for the paired bone marrow samples**

| No. | Sex | Age(years) | The date of specimens collection at newly diagnosis | Immunotype | Cytogenetic abnormality | Fusion gene | Prognosis |
| --- | --- | --- | --- | --- | --- | --- | --- |
| 1 | F | 9 | 2007.06 | Common B cell | t(1;19) | *E2A-PBX1* | Remission |
| 2 | F | 3 | 2008.05 | Common B cell | t(12;21) | *TEL-AML1* | Remission |
| 3 | M | 3 | 2009.04 | Common B cell | t(12;21) | *TEL-AML1* | Remission |
| 4 | F | 3 | 2008.06 | Common B cell | t(12;21) | *TEL-AML1* | Remission |
| 5 | M | 5 | 2005.11 | Common B cell | t(12;21) | *TEL-AML1* | Remission |
| 6 | M | 7 | 2007.08 | Common B cell | t(1;19) | *E2A-PBX1* | Remission |
| 7 | M | 3 | 2008.04 | Pre-B cell | t(12;21) | *TEL-AML1* | Remission |
| 8 | M | 3 | 2011.06 | Common B cell | － | － | Remission |
| 9 | M | 6 | 2011.01 | Common B cell | － | － | Remission |
| 10 | M | 6 | 2011.02 | Common B cell | － | － | Remission |
| 11 | M | 4 | 2006.08 | Common B cell | t(12;21) | *TEL-AML1* | Remission |
| 12 | F | 3 | 2006.08 | Common B cell | t(12;21) | *TEL-AML1* | Remission |
| 13 | F | 3 | 2006.06 | Common B cell | t(12;21) | *TEL-AML1* | Remission |
| 14 | M | 4 | 2006.06 | Common B cell | t(12;21) | *TEL-AML1* | Remission |
| 15 | M | 3 | 2006.02 | Common B cell | － | － | Remission |
| 16 | M | 2 | 2006.01 | Common B cell | t(12;21) | *TEL-AML1* | Dead |
| 17 | M | 1 | 2004.11 | Mature B cell | － | － | Remission |
| 18 | F | 3 | 2005.11 | Common B cell | － | － | Remission |
| 19 | M | 2 | 2005.08 | Common B cell | － | － | Remission |
| 20 | F | 3 | 2007.03 | Common B cell | t(12;21) | *TEL-AML1* | Remission |
| 21 | M | 6 | 2007.10 | Common B cell | t(1;19) | *E2A-PBX1* | Remission |
| 22 | M | 4 | 2007.09 | Common B cell | t(12;21) | *TEL-AML1* | Remission |
| 23 | M | 3 | 2007.07 | T cell | del(1) | *SIL-TAL1* | Remission |
| 24 | M | 4 | 2007.12 | Common B cell | t(12;21) | *TEL-AML1* | Remission |
| 25 | M | 4 | 2007.11 | Common B cell | － | － | Remission |
| 26 | M | 8 | 2007.09 | Common B cell | － | － | Remission |
| 27 | M | 4 | 2007.10 | Common B cell | t(12;21) | *TEL-AML1* | Remission |
| 28 | F | 2 | 2007.09 | Common B cell | t(12;21) | *TEL-AML1* | Remission |
| 29 | M | 7 | 2007.05 | Common B cell |  | － | Remission |
| 30 | F | 6 | 2007.05 | Common B cell | t(12;21) | *TEL-AML1* | Remission |
| 31 | M | 3 | 2007.05 | Common B cell | － | － | Remission |
| 32 | M | 5 | 2007.06 | Common B cell | － | － | Remission |
| 33 | M | 5 | 2007.05 | Common B cell | － | － | Remission |
| 34 | F | 8 | 2007.03 | pro-B cell | － | － | Remission |
| 35 | M | 2 | 2007.01 | Common B cell | － | － | Remission |
| 36 | M | 7 | 2007.02 | Common B cell | － | － | Remission |
| 37 | F | 2 | 2007.01 | Common B cell | － | － | Remission |
| 38 | M | 1 | 2006.12 |  | － | － | Remission |
| 39 | M | 2 | 2007.10 | Common B cell | － | － | Remission |
| 40 | M | 6 | 2008.04 | Common B cell | t(9;22) | *BCR-ABL* | Remission |
| 41 | M | 7 | 2008.10 | Common B cell | t(9;22) | *BCR-ABL* | Remission |
| 42 | F | 2 | 2007.08 | Common B cell | t(12;21) | *TEL-AML1* | Remission |
| 43 | M | 5 | 2007.11 | Common B cell | t(1;19) | *E2A-PBX1* | Remission |
| 44 | F | 2 | 2008.06 | T cell | del(1) | － | Remission |
| 45 | F | 3 | 2008.06 | Pro-B cell | t(12;21) | *TEL-AML1* | Remission |
