# Supplementary material for: Correlation of SRSF1 and PRMT1 expression with clinical status of pediatric acute lymphoblastic leukemia
Source: J Hematol Oncol. 2012 Jul 27;5:42. doi: 10.1186/1756-8722-5-42 (PMC3459738; doi:10.1186/1756-8722-5-42)
Supplement: Additional file 2 — Table S2. Clinical features of the pediatric acute leukemia cases for the unpaired bone marrow samples. Detailed characteristics of eight pediatric patients for the unpaired samples are shown here. [file 1756-8722-5-42-S2.docx]

**Additional file 2. Clinical features of the pediatric acute leukemia cases for the unpaired bone marrow samples**

| No. | Sex | Age(years) | The date of specimens collection at newly diagnosis | Immunotype | Fusion gene | Prognosis |
| --- | --- | --- | --- | --- | --- | --- |
| 1 | F | 10 | 2008.11 | Common B cell | － | Remission |
| 2 | M | 5 | 2004.08 | T cell | － | Remission |
| 3 | M | 2 | 2008.09 | Common B cell | － | Remission |
| 4 | F | 7 | 2006.09 | Common B cell | － | Remission |
| 5 | M | 6 | 2008.11 | Common B cell | － | Remission |
| 6 | M | 6 | 2006.09 | Common B cell | － | Remission |
| 7 | F | 10 | 2009.02 | Common B cell | － | Remission |
| 8 | M | 5 | 2006.06 | Common B cell | *TEL-AML1* | Remission |
