# Supplementary material for: Correlation of SRSF1 and PRMT1 expression with clinical status of pediatric acute lymphoblastic leukemia
Source: J Hematol Oncol. 2012 Jul 27;5:42. doi: 10.1186/1756-8722-5-42 (PMC3459738; doi:10.1186/1756-8722-5-42)
Supplement: Additional file 3 — Table S3. Clinical features of pediatric acute leukemia cases for the relapsed bone marrow samples. Detailed characteristics of four relapsed patients are shown here. [file 1756-8722-5-42-S3.docx]

**Additional file 3.** **Clinical features of the pediatric acute leukemia cases for the relapsed bone marrow samples**

| No. | Sex | Age(years) | The date of specimens collection at newly diagnosis | The date of specimens collection at complete remission | The date of specimens collection at relapse | Immunotype | Fusion gene | Prognosis |
| --- | --- | --- | --- | --- | --- | --- | --- | --- |
| 1 | M | 7 months | 2002.10 | 2003.10 | 2007.01 | Common B cell | *MLL* | Dead |
| 2 | M | 15 | 2007.07 | 2008.02 | 2008.12 | Common B cell | － | Dead |
| 3 | M | 10 | 2005.05 | 2006.04 | 2008.01 | Common B cell | － | Dead |
| 4 | M | 9 | 2006.04 | 2006.10 | 2007.05 | Common B cell | － | Dead |
