# Supplementary material for: Correlation of SRSF1 and PRMT1 expression with clinical status of pediatric acute lymphoblastic leukemia
Source: J Hematol Oncol. 2012 Jul 27;5:42. doi: 10.1186/1756-8722-5-42 (PMC3459738; doi:10.1186/1756-8722-5-42)
Supplement: Additional file 4 — Doc1. Bio-informatics methods for the heat map of mRNA level ofSFRS1. Detailed methods of bio-informatics analysis of mRNA level of SFRS1 are shown here [18,19][36]. [file 1756-8722-5-42-S4.docx]

**Additional file 4. Bio-informatics methods for the heat map of mRNA level of *SFRS1***

Based on the dataset of gene expression profiles of 100 pediatric ALL patients ^[^[^18^](#_ENREF_18)^,^ [^19^](#_ENREF_19)^]^, we employed an NP (Negative and positive correlations) network ^[^[^36^](#_ENREF_36)^]^ to integrate gene expression profiles with the protein-protein interaction (PPI) network, to obtain tightly correlated gene clusters. We first searched for PPIs between genes whose expression profiles were either correlated or anti-correlated with the top 5% and bottom 5% Pearson correlation coefficient (PCC) values. We then hierarchically clustered the genes in this subnet work and delimited the cluster based on a ratio of intra-cluster transcriptionally anti-correlated to correlated PPIs of 0.01. HPRD, downloaded on September 13, 2005 was used as the PPI dataset.

For the clusters based on raw data, Paired Student’s t-test of all the genes in a cluster between the control and disease samples were used to determine significantly up- or down-regulated clusters (*p*<0.025 or *p*>0.975). Another set of clusters based on log2 transformed data is also calculated using the NP method.

A separate gene list of up- or down-regulated clusters were put together from genes that are up- or down-regulated in more than half (50/100) of ALL patient samples by at least 1.5 fold in each sample. We then used the intersection of the genes in this list with the genes in significantly up- or down-regulated clusters and gene clusters,s based on log2 transformed data described above as our high confidence up- or down-regulated gene sets. We also performed motif analysis on each of the two gene sets. Finally, 36 up-regulated genes (Figure 1A) and 48 down-regulated genes (not shown) were screened out.
